# Cardioprotective Solutions Exposure For 1 Hour in Hypoxia and Low Temperatures Affects Vascular Reactivity Differently

**DOI:** 10.21470/1678-9741-2020-0320

**Published:** 2021

**Authors:** Priscila Rossi de Batista, Dalton Valentim Vassallo, Maylla Ronacher Simões, Melchior Luiz Lima

**Affiliations:** 1 Department of Physiological Sciences, Federal University of Espírito Santo, Vitória, ES, Brazil.; 2 Centro de Ciências da Saúde de Vitória-EMESCAM, Vitória, ES, Brazil.

**Keywords:** Vasoconstriction, Vasoconstrictor Agents, Hypoxia, Endothelium, Temperature, Phenylephrine

## Abstract

**Introduction:**

Heart preservation benefits cardiac performance after operations decreasing morbidity but the contribution of the vascular reactivity has been neglected.

**Objective:**

We evaluated whether cardioprotective solutions, Krebs-Henseleit (KH), Bretschneider-HTK (BHTK), St. Thomas No. 1 (STH-1), and Celsior (CEL), affect vascular reactivity.

**Methods:**

Aortic rings from Wistar rats were used in two protocols. First, the rings were exposed to BHTK, STH-1 or CEL for 1 hour of hypoxia at 37 °C. Second, the rings were exposed to 10 °C or 20 °C for 1 hour under hypoxia. After treatment, the rings were immersed in KH at 37 °C, endothelial integrity was tested and concentration-response curves to phenylephrine were performed.

**Results:**

In the first protocol, the solutions did not damage the endothelium; CEL and BHTK reduced KCl-induced contractions but not STH-1; only CEL and BHTK reduced vascular reactivity; there was a positive correlation between R_max_ and KCl concentration. At 20 °C, 1 hour under hypoxia, the solutions produced similar KCl-induced contractions without endothelial damage. CEL, BHTK and STH-1 decreased vascular reactivity. At 10 °C, STH-1 increased reactivity but CEL and BHTK decreased. After 1 hour under hypoxia in CEL or BHTK solutions, reactivity was similar at different temperatures. At 20 °C, endothelial damage after exposure to STH-1 produced more vasoconstriction than CEL and BHTK. However, at 10 °C, endothelial damage after CEL and BHTK exposure elicited more vasoconstriction while STH-1 showed a small vasoconstrictor response, suggesting endothelial damage.

**Conclusion:**

STH-1 decreased reactivity at 20 °C and increased at 10 °C. CEL promoted greater endothelial modulation at 10 °C than at 20 °C, while STH-1 promoted higher modulation at 20 °C than at 10 °C. Vascular tone was reduced by CEL and BHTK exposure, also depending on the KCl concentration.

**Table:** 

Abbreviations, acronyms & symbols				
**ACh**	**= Acetylcholine chloride**		**KH**	**= Krebs-Henseleit**
**ATP**	**= Adenosine triphosphate**		**KH_2_PO_4_**	**= Monopotassium phosphate**
**AUC**	**= Area under the curve**		**mM**	**= Millimolar**
**ANOVA**	**= Analysis of variance**		**MgSO_4_**	**= Magnesium sulfate**
**BHTK**	**= Bretschneider-HTK**		**NaCl**	**= Sodium chloride**
**CaCl_2_**	**= Calcium chloride**		**NaHCO_3_**	**= Sodium bicarbonate**
**CEL**	**= Celsior**		**PHE**	**= Phenylephrine**
**CO_2_**	**= Carbon dioxide**		**R_max_**	**= Maximal response**
**dAUC**	**= Differences in areas under the curves**		**SEM**	**= Standard error of the mean**
**EDTA**	**= Ethylenediaminetetraacetic acid**		**STH-1**	**= St. Thomas No. 1**
**HTK**	**= Histidine-tryptophan-ketoglutarate**		**µM**	**= Micrometer**
**KCl**	**= Potassium chloride**			

## INTRODUCTION

Myocardial protection has great relevance for the advances in heart transplantation^[1]^. Cardioprotection refers to strategies used to attenuate or prevent myocardial dysfunction during or after cardiac surgery. To achieve this goal, cardioprotective and cardioplegic solutions have been used. The mechanism of a cardioprotective solution is based upon three principles: a) Hypothermic stop of the metabolism; b) A physical and biochemical environment to maintain viable tissue structure during the hypothermic stop of the metabolism; and c) Minimizing the effects of the reperfusion injury^[2]^.

The main purpose of the composition of preservation solutions is to induce a fast depolarization of the cardiac myocyte membrane by reducing the transmembrane K^+^ gradient^[3]^. This procedure stops the mechanical and electrical activity of the heart. Saline solutions with composition similar to the extracellular fluid such as Ringer’s lactate and Hartmann were initially used for myocardial protection but were considered inadequate to maintain the myocardial protection and its function^[4]^.

To improve contractile function after periods of ischemia, hypothermic procedures have been used^[5-7]^. Hypothermia reduces energy consumption by protecting cellular metabolism and improving resistance to ischemia during cardioplegic cardiac arrest^[8]^. The increase in the supply and energy demand ratio during ischemia is generally attributed to hypothermic protection and hypothermia also provides an important reduction of oxidative stress induced by ischemia and reperfusion^[9]^.

So far, cardiac protection for transplantation has been focused mainly on the cardiac mechanical activity but studies about the effects of solutions used on vascular reactivity have been neglected. Some focus was given to the coronary vessels^[10,11]^ but not to the systemic vasculature and under different temperatures. If these protective solutions damage the vessels, they can be harmful to the heart blunting its mechanical recovery and increasing afterload during ventricular ejection. Therefore, we aimed to study how preservation solutions used in cardiac transplantation affect vascular reactivity. Our attempt was to investigate whether the Krebs-Henseleit (KH), Bretschneider-HTK (BHTK), St. Thomas No. 1 (STH-1) and Celsior (CEL) solutions might affect the vascular reactivity of aortic rings, a conductance vessel, submitted to 1 hour of hypoxia, and if 1 hour of hypoxia at 10 and 20 °C alters vascular reactivity, producing or avoiding endothelial damage. Results will enable us to select which solutions induce non-deleterious changes for further investigation.

## METHODS

### Animals and Treatment

Male Wistar rats (260-300 g, N=64) were used for these studies. The care and use of laboratory animals were in accordance with the National Institutes of Health guidelines, and all experiments were conducted in accordance with the guidelines for biomedical research as stated by the Brazilian Societies of Experimental Biology and were approved by the Institutional Ethics Committee of the Health Sciences Center of Vitória (CEUA-EMESCAM 004/2007). All rats had free access to water and were fed with rat chow *ad libitum*. The rats were anesthetized with pentobarbital (35 mg/kg, i.p.) and euthanized by exsanguination. The thoracic aortas were carefully dissected, placed in aerated KH solution and fat and connective tissue were removed. For vascular reactivity experiments, the aortas were divided into cylindrical segments 4 mm long.

Two protocols were performed. In the first protocol, the rings were immersed in KH, BHTK, STH-1 and CEL solutions for 1 hour of hypoxia, at 37 °C. In the second protocol, the rings were immersed in BHTK, STH-1 and CEL solutions, at 10 or 20 °C for 1 hour of hypoxia, except for the KH solution, in which the rings were kept at 37 °C, and gassed with 95% O_2_-5% CO_2_ pH 7.4.

### Vascular Reactivity Measurements

#### Hypoxia Protocol

This protocol was performed first to define if the selected cardioprotective solutions could cause any vascular harm. After anaesthesia, the descending thoracic aorta was removed and placed in a Petri dish immersed in KH solution. The rings were immediately placed in the solutions of interest (CEL, BHTK, or STH-1) without aeration with a carbogenic mixture for 1 hour at 37 °C. After 1 hour of hypoxia, the aortic segments were mounted between two parallel wires in organ baths containing KH solution (in mM: 124 NaCl, 4.6 KCl, 2.5 CaCl_2_, 1.2 MgSO_4_, 1.2 KH_2_PO_4_, 0.01 EDTA, 23 NaHCO_3_) at 37 °C and gassed with 95% O_2_-5% CO_2_ (pH 7.4). The arterial segments were stretched to an optimal resting tension of 1 g. The tension was recorded using a force transducer (TSD125C, CA, USA) connected to an acquisition system (MP100A, BIOPAC System, Inc., Santa Barbara, USA). After a 45 minutes equilibration period, all aortic rings were initially exposed twice to 75 mM KCl. The first exposure checks their functional integrity, and the second exposure assesses the maximal tension. Next, endothelial integrity was tested with acetylcholine (ACh, 10 µM) in segments previously contracted with phenylephrine (1 µM). A relaxation ≥90% was considered indicative of the functional integrity of the endothelium. After a washout period (30 min), concentration-response curve for phenylephrine (0.1 nM-30 mM) was performed and the tension was measured once a plateau was attained.

#### Temperature Protocols

After anaesthesia, the descending thoracic aorta was removed and placed in a Petri dish immersed in KH solution. Then, the rings were immediately placed in the solutions of interest (CEL, BHTK, or STH-1), without carbogenic mixture, for 1 hour at a temperature of 20 or 10 °C; at the end of this period, the rings were placed in the system under the normal experimental condition, KH solution, with carbogen, at a constant temperature of 37 °C (control condition).

Control Group: After cutting, the rings were placed in the control condition described above during the same period (1 h). Thus, all groups were submitted to the same experimental protocols described above: a) Viability test of vascular smooth muscle and endothelium; b) Evaluation of vascular reactivity from concentration-response curves to phenylephrine (PHE, 10-10 to 10-4 M); c) This same evaluation of vascular reactivity was performed in the absence of the endothelium (E), after mechanical injury of the rings before being placed in the system, to comparatively study the endothelium participation.

#### Drugs and Reagents

L-phenylephrine hydrochloride, ACh and sodium pentobarbital were purchased from Sigma-Aldrich (St. Louis, USA). Salts and reagents used were of analytical grade from Sigma-Aldrich and Merck (Darmstadt, Germany).

### Statistical Analyses

Results are expressed as the mean±SEM of the number of rats studied; differences were analysed using Student’s t-test or one or two-way ANOVA followed by a Bonferroni test. A *P*<0.05 was considered significant. Contractile responses to phenylephrine were expressed in absolute values. For each concentration-response curve, the maximal response (R_max_) and the concentration of agonist that produced 50% of the maximal response (log EC_50_) were calculated using non-linear regression analysis (GraphPad Prism, GraphPad Software, Inc., San Diego, CA). The sensitivities of the agonists were expressed as pD_2_ (-log EC_50_). To compare the effects of different solutions and temperatures, some results were expressed as differences in the areas under the concentration-response curves (dAUC) in both the control and experimental groups. AUCs were calculated from individual concentration-response curve plots: the differences are expressed as the percentage of the control AUC.

## RESULTS

**Fig. 1:**
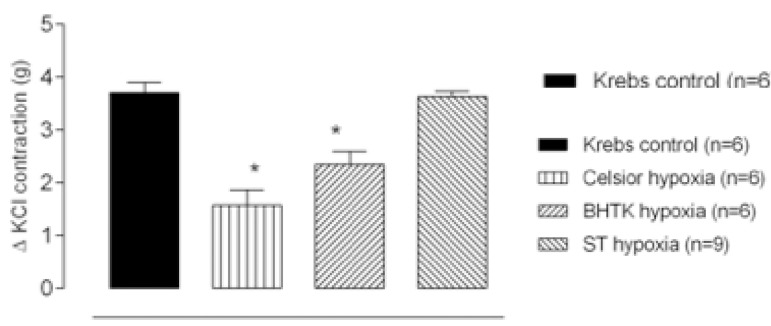
Contractile response to potassium chloride (KCl-75 mM) in isolated aortic rings after 1 hour of hypoxia at 37 °C in Krebs-Henseleit (Krebs control), Celsior, Bretschneider-HTK (BHTK) or St. Thomas (ST) solutions. Results expressed as mean±SEM. *P<0,05 vs. Krebs-Henseleit (control), one-way ANOVA, Turkey test. The numbers in parentheses indicate the number of specimens used.

Our first attempt was to investigate whether 1 hour of hypoxia at 37 °C would affect the vascular contraction induced by 75 mM of KCl. Figure 1 shows that, after hypoxia, KCl-induced contractions were different among the solutions used. Krebs and STH-1 did not change KCl-induced contraction, but CEL and BHTK solutions reduced the contractions of aortic rings.

However, at 20 and 10 °C, all groups exposed to STH-1, CEL and BHTK solutions presented, after 1 hour of hypoxia, similar KCl-induced contractions (in g: CEL at 20 °C 3.29±0.2, at 10 °C 3.16±0.2; STH-1 at 20 °C 32±0.2, at 10 °C 3.77±0.1; BHTK at 20 °C 3.36±0.2, at 10 °C 3.38±0.2). In addition, hypoxia for 1 hour, in both protocols, did not affect the functional endothelial integrity. The relaxation induced by ACh (10 µM) was preserved in segments exposed to different solutions, previously contracted with phenylephrine (1 µM) (results not shown).

**Fig. 2:**
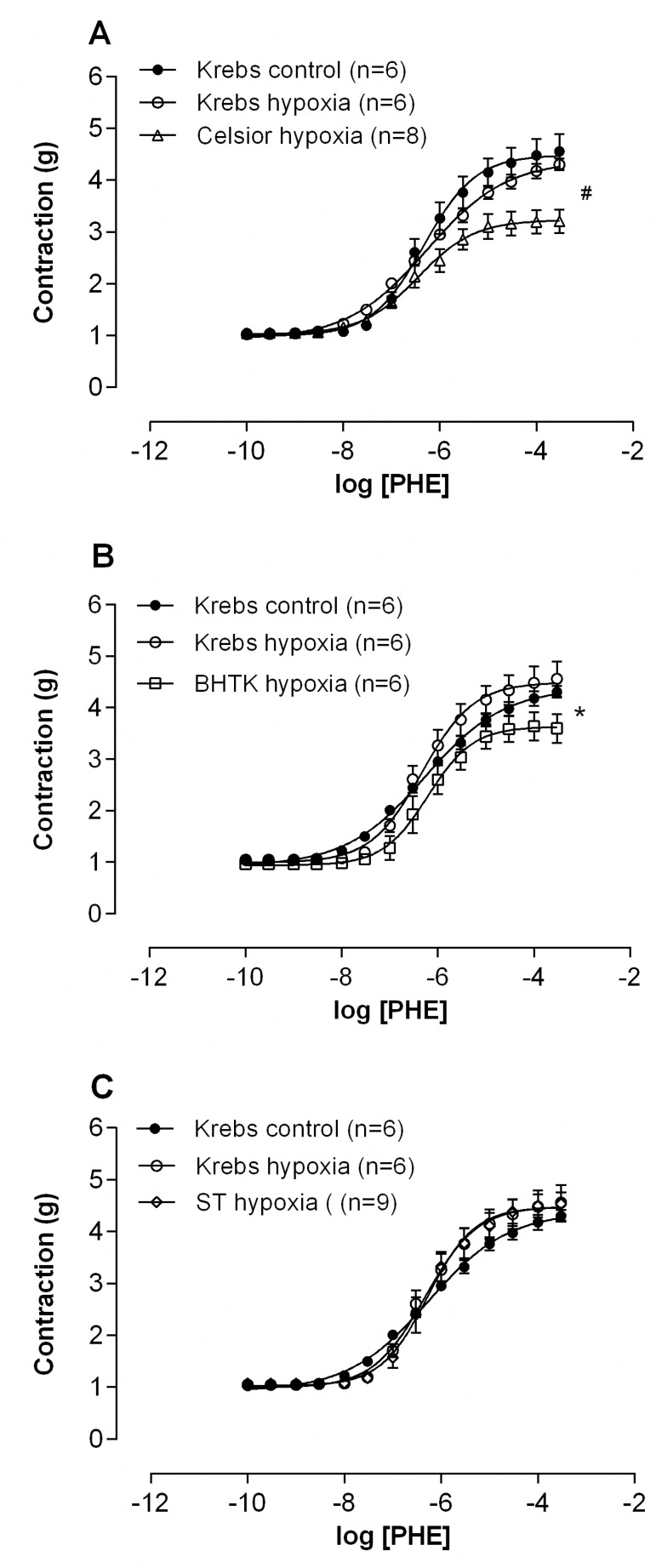
Concentration-response curves to phenylephrine (PHE) in isolated aortic rings of Wistar rats after incubation for 1 hour in hypoxia with Celsior (A), Bretschneider-HTK (BHTK) (B) and St. Thomas (ST) (C) solutions, compared to the Krebs control solution, at 37 °C and after 1 hour of hypoxia. Results expressed as mean±SEM. *P<0.05 for comparison of Rmax vs. control Krebs, t-test. The numbers in parentheses indicate the number of specimens used.

Hypoxia at 37 °C did not change the capacity of aortic rings, previously exposed to preservation solutions and then incubated with Krebs, to contract in response to phenylephrine (hypoxia at 37 °C, Figure 2). However, concentration-response curves to phenylephrine showed a reduction in R_max_ after 1 hour of hypoxia under CEL and BHTK solutions. Concerning STH-1 solution, aortic rings showed vascular reactivity to phenylephrine similar to Krebs control (hypoxia at 37 °C, Figures 2A, B and C).

**Fig. 3:**
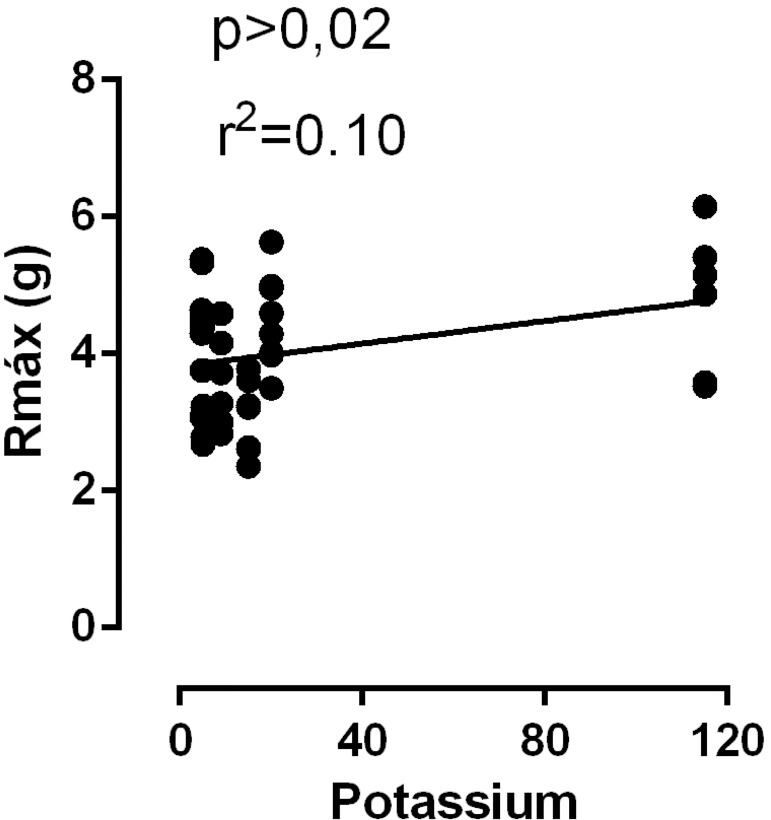
Correlation between Rmax and the potassium concentration of the studied solutions Krebs-Henseleit (Krebs control), Celsior, Bretschneider-HTK and St. Thomas-1. P<0.02.

As mentioned above, CEL and BHTK solutions reduced the vascular reactivity of aortic rings to phenylephrine. To understand these results, we also investigated a putative relationship between R_max_ and the composition of the solutions. There was no correlation between calcium concentratio n and R_max_ (results not shown). However, there was a positive correlation between R_max_ and the potassium concentration of the solutions (hypoxia at 37 °C, Figure 3), suggesting that the maintenance of a reduced vascular tone depends on the smaller amount of potassium in the solutions.

**Fig. 4:**
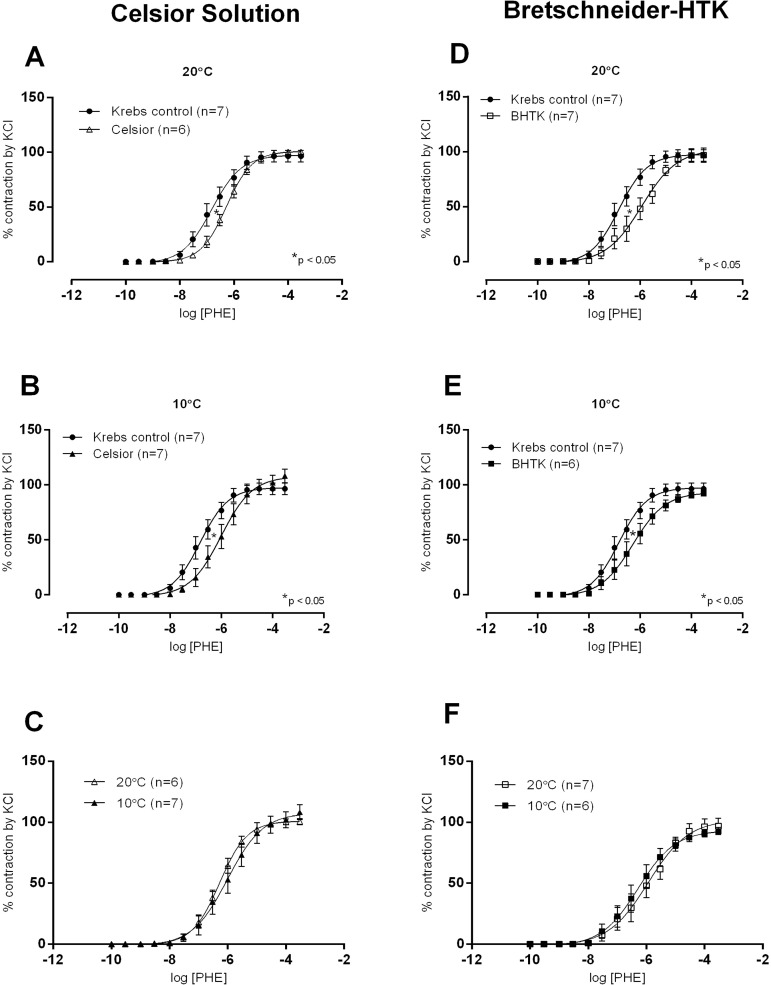
Concentration-response curves to phenylephrine (PHE) in isolated aortic rings of Wistar rats at 37 °C in Krebs-Henseleit (Krebs control) solution after incubation for 1 hour in hypoxia at 20 °C or 10 °C with Celsior (A and B) or Bretschneider-HTK (BHTK) (D and E) solutions in pD2 and Rmax values. In C (Celsior) and F (BHTK), comparisons of concentration-response curves at 20 °C or 10 °C. Results were expressed as mean±SEM. *P<0.05 vs. Krebs control solution, t-test for Rmax and pD2. The numbers in parentheses indicate the number of specimens used.

Figure 4 shows the concentration-response curves to phenylephrine and the effects of exposing aortic rings to CEL solution at 10 and 20 °C compared to control Krebs solution at 37 °C. Sensitivity was reduced at both temperatures (Figures 4A and B) but without differences when compared between them (Figure 4C). However, concentration-response curves to phenylephrine, at both temperatures, did not show changes of R_max_. Regarding the BHTK solution, a similar behaviour as CEL was observed (Figures 4D, E and F). These findings suggest that, if exposed to vasoconstrictors, these vessels would preserve a better flow by developing smaller contractions.

**Fig. 5:**
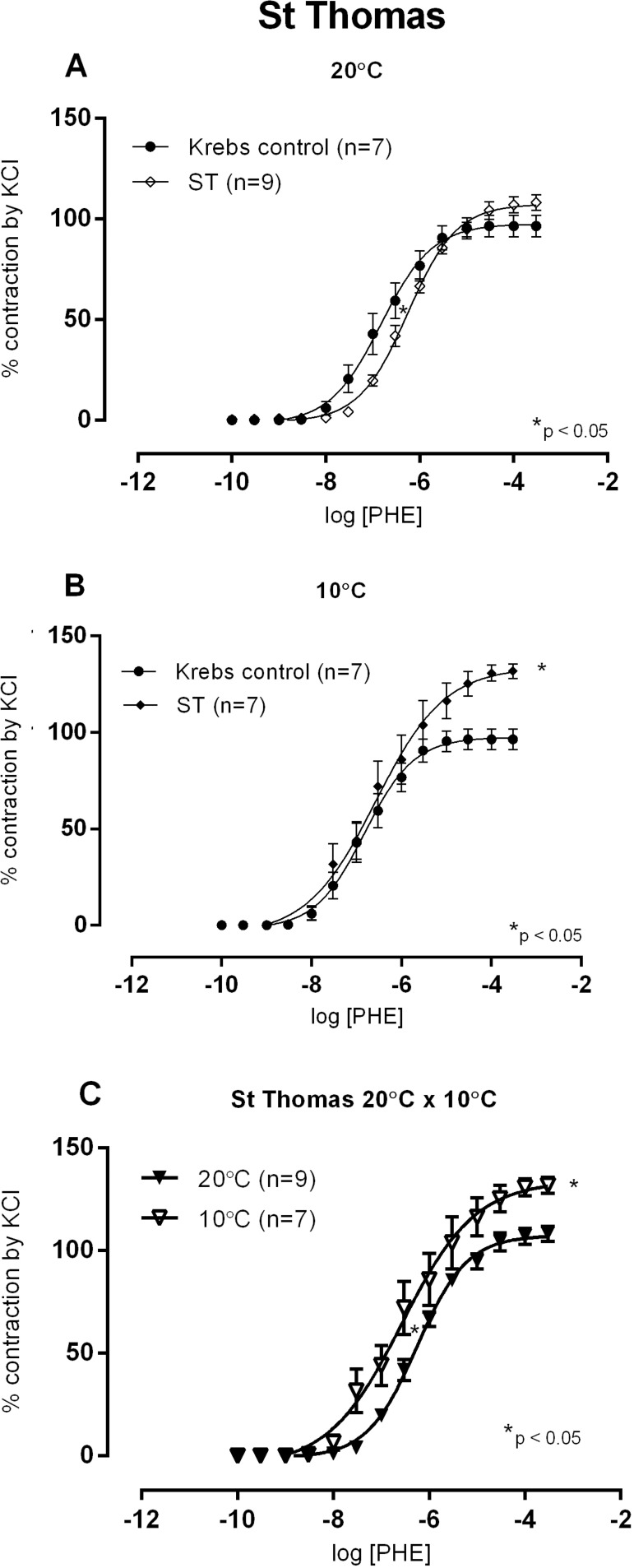
Concentration-response curves to phenylephrine (PHE) in isolated aortic rings of Wistar rats at 37 °C in Krebs-Henseleit (Krebs control) solution after incubation for 1 hour in hypoxia at 20 °C (A) or 10 °C (B) of Rmax or pD2 of St. Thomas (ST) solution, compared to Krebs control solution, at 37 °C. In (C), comparison between concentration-response curves to PHE at 20 °C or 10 °C. Results expressed as mean±SEM. *P<0.05 vs. CT (control); t-test for Rmax and pD2. The numbers in parentheses indicate the number of specimens used.

Regarding the 10 °C and 20 °C protocol, STH-1 solution showed an increased sensitivity of the vascular reactivity to phenylephrine at 20 °C when compared to control Krebs solution at 37 °C, but not at 10 °C (10 and 20 °C protocol, Figures 5A and B). Moreover, R_max_ increased after exposure to STH-1 solution at 10 °C but not at 20 °C. However, comparing concentration-response curves to phenylephrine at both temperatures, R_max_ and sensitivity were higher at 10 °C (10 and 20 °C protocol, Figure 5C). These findings suggest that, if exposed to vasoconstrictors, these vessels would react more, increasing vasoconstriction and the afterload during ventricular ejection.

**Fig. 6:**
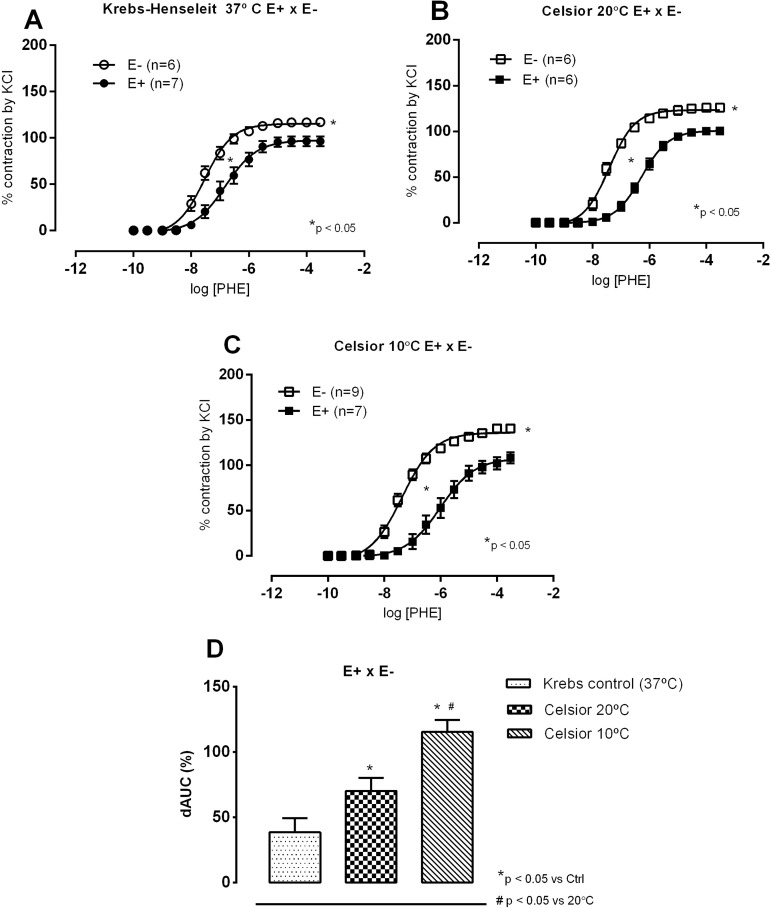
Concentration-response curves to phenylephrine (PHE) in the presence (E+) and absence of endothelium (E-) in isolated aortic rings of Wistar rats at 37 °C in Krebs-Henseleit solution, after incubation for 1 hour in hypoxia. In Krebs solution at 37 °C (A) and at 20 °C (B) or 10 °C (C) with the Celsior solution; comparison of endothelial modulation by calculating the difference of area below the curve (dAUC), in % (D). Results expressed as mean±SEM. In A, B and C, P<0.05 for comparisons of Rmax and pD2 at 37 °C, 20 °C or 10 °C, t-test. In D, *P<0.05 for comparisons of Krebs control vs. CEL at 20 °C or 10 °C, and #P<0.05 for comparisons of Krebs control vs. Celsior at 20 °C or 10 °C, one-way ANOVA, Tukey test. The numbers in parentheses indicate the number of specimens used.

**Fig. 7:**
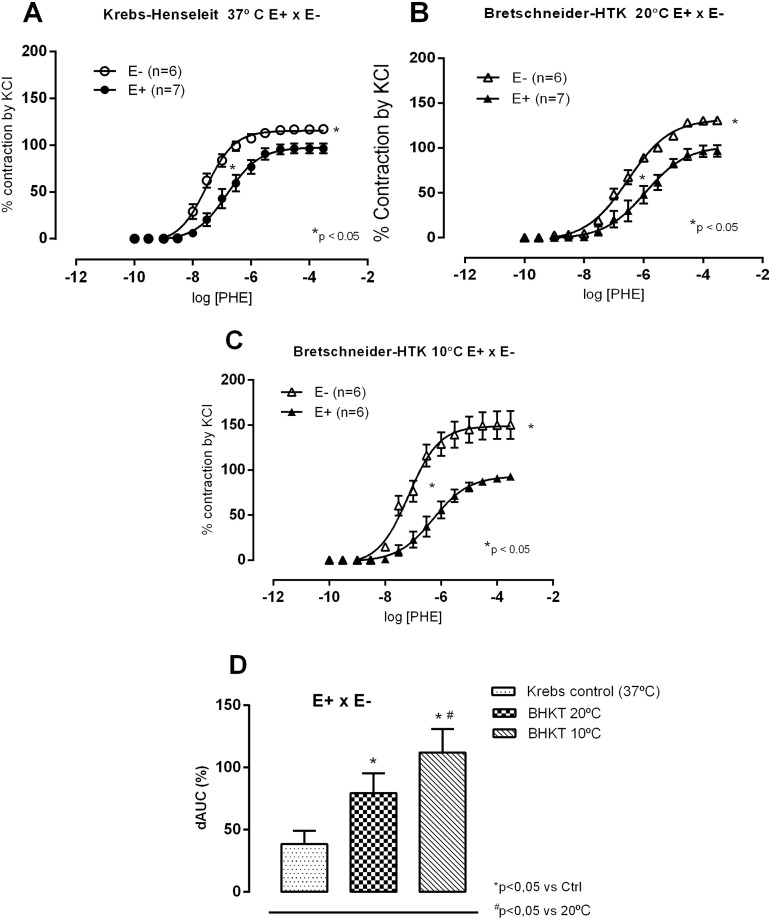
Concentration-response curves to phenylephrine (PHE) in the presence (E+) and absence of endothelium (E-) in isolated aortic rings of Wistar rats at 37 °C in Krebs-Henseleit solution, after incubation for 1 hour in hypoxia. In Krebs solution at 37 °C (A) and at 20 °C (B) or 10 °C (C) with the Bretschneider-HTK solution; comparison of endothelial modulation by calculating the difference of area below the curve (dAUC), in % (D). Results expressed as mean±SEM. In A, B and C, P<0.05 for comparisons of Rmax and pD2 at 20 °C or 10 °C, t-test. In D, *P<0.05 for comparisons of CT vs. Bretschneider-HTK at 37 °C, 20 °C or 10 °C, and #P<0.05 for comparisons of Krebs control vs. BHTK at 20 °C or 10 °C, one-way ANOVA, Tukey test. The numbers in parentheses indicate the number of specimens used.

To understand these results, we also investigated the role of the endothelium on such responses. With the Krebs solution, as expected, the endothelial removal increased contractile responses to phenylephrine. Regarding CEL (10 and 20 °C protocol, Figure 6) and BHTK solutions (10 and 20 °C protocol, Figure 7), endothelial removal increased both R_max_ and sensitivity at both temperatures but these changes were enhanced at 10 °C when compared to 20 °C. These findings suggest that, if for some other reason the endothelium is damaged, an increased vasoconstriction will occur at 10 °C. These results are highlighted in Figures 6D and 7D, that show dAUCs comparisons of concentration-response curves to phenylephrine under control conditions (Krebs at 37 °C) and after exposure to CEL and BHTK solutions.

**Fig. 8:**
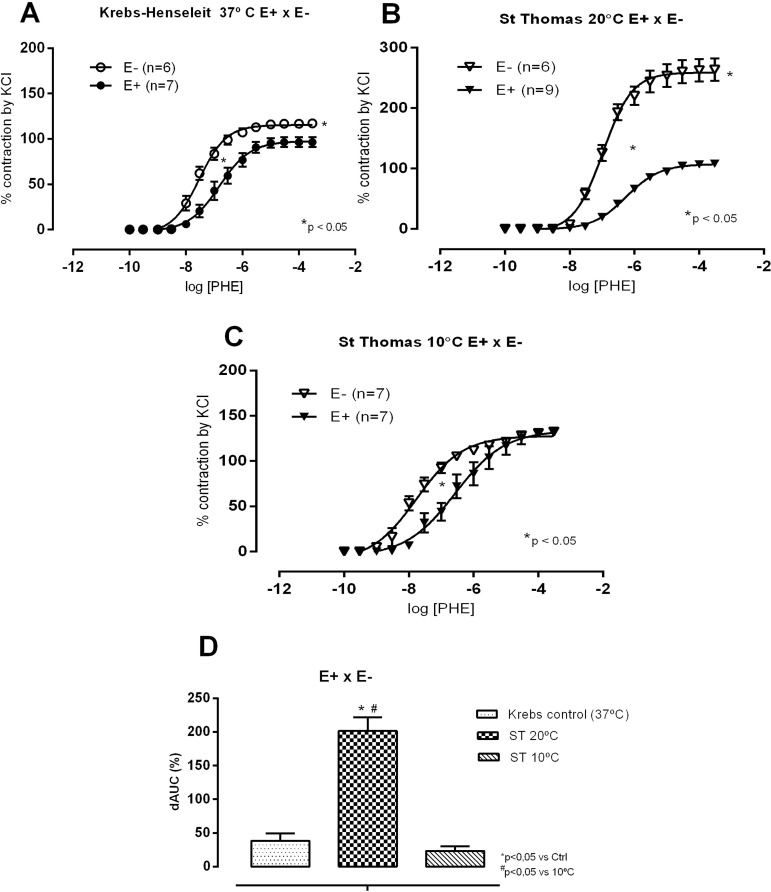
Concentration-response curves to phenylephrine (PHE) in the presence (E+) and absence of endothelium (E-) in isolated aortic rings of Wistar rats at 37 °C in Krebs-Henseleit solution, after incubation for 1 hour in hypoxia. In Krebs solution at 37 °C (A) and at 20 °C (B) or 10 °C (C) with St. Thomas solution; comparison of endothelial modulation by calculating the difference of area below the curve (dAUC), in % (D). Results expressed as mean±SEM. In (A) and (B), P<0.05 for comparisons of Rmax and pD2 at 37 °C, 20 °C or 10 °C, t-test. In (D), *P<0.05 for comparisons of Krebs control vs. St. Thomas at 20 °C or 10 °C, and #P<0.05 for comparisons of CT vs. St. Thomas at 20 °C or 10 °C, one-way ANOVA, Tukey test. The numbers in parentheses indicate the number of specimens used.

However, when investigating the STH-1 solution (10 and 20 °C protocol, Figures 8A and B), we observed that the reactivity to phenylephrine was much higher at 20 °C than at 10 °C. In Figure 8D, dAUCs show that this behaviour is enhanced when comparing STH-1 solution exposure to control conditions C and to 10 °C. These findings suggest that, if the rings are exposed to 10 °C with a damaged endothelium, as the temperature increases, the reactivity to vasoconstrictors will increase dramatically, compromising left ventricular ejection by an increased afterload.

## DISCUSSION

Our results showed that the cardioprotective solutions used (Krebs, CEL, BHTK, and STH-1) administered at 37 °C for 1 hour of hypoxia did not damage the endothelium. However, Ringer, CEL and BHTK exposure reduced KCl-induced contractions. The cardioprotective solutions used (CEL, BHTK and STH-1), administered at 10 and 20 °C for 1 hour in hypoxia, also did not alter the endothelial function but the KCl-induced contractions were similar.

When performing cardiac arrest for several purposes, myocardial ischemia takes place decreasing the production of adenosine triphosphate (ATP) and myocardial function. Prolonged ischemic periods may compromise myocardial viability even with methods of preservation. Thus, adequate myocardial preservation is a critical measure to ensure the viability of the heart in procedures performed with prolonged ischemic time^[12]^. Temperature reduction is frequently used for such purpose^[13]^ but so far there is no gold standard for myocardial protection^[14]^. Hypothermia is a standard strategy and, according to Cleveland et al.^[15]^, is the most important factor in myocardial protection.

Myocardial preservation studies have been the main goal to achieve good results and are mainly focused on the cardiac mechanical activity^[5-7]^. Moreover, studies regarding the effects of preservation solutions on vascular reactivity have been neglected. Therefore, if protective solutions damage the endothelium, they could be harmful to the heart, impairing blood flow and its mechanical recovery, increasing the vascular tone of conductance arteries and, consequently, the afterload against which the left ventricle has to eject.

We previously investigated the effects of cardioplegic or cardioprotective solutions, after 1 hour of hypoxia, at 37 °C, to select which solutions would be suitable to be evaluated at 10 °C and 20 °C. KH, BHTK, STH-1, and CEL solutions were investigated covering a range of protective profiles such as different levels of potassium to keep the cell membrane depolarized and the myocardium arrested, free radicals scavengers, non-permeants to maintain osmotic pressure and to prevent edema, energy substrates, and buffers to prevent acidosis^[16]^. Our results showed that CEL and BHTK solutions, at such conditions (1 hour of hypoxia at 37 °C), reduced the vascular reactivity to phenylephrine.

Moreover, at 37 °C, the concentration-response curves to phenylephrine showed a reduction of R_max_ after 1 hour of hypoxia under CEL and BHTK solutions, suggesting a reduction in vascular tone. This reduction was correlated with the reduction in the potassium concentration of the solutions, since CEL and BHTK solutions have a lower KCl concentration. Moreover, there was a positive correlation between R_max_ and the potassium concentration of the solutions, suggesting that the maintenance of a reduced vascular tone depended on the amount of potassium in the solutions used. In agreement, a recent study demonstrated that the CEL solution, which has a smaller KCl concentration, is the best for myocardial protection in isolated hearts submitted to cold static ischemia^[17]^.

Other studies on myocardial protection have shown benefits of the CEL and BHTK solutions^[18]^. Pereda et al.^[19]^ compared the performance of CEL with STH-1 solutions, demonstrating that they were not significantly different. Based on these findings, we investigated CEL, BHTK and STH-1 solutions after 1 hour of hypoxia at 10 °C and 20 °C, and compared their effects with KH solution under control conditions, 37 °C and no hypoxia. Concentration-response curves to phenylephrine, after exposure to 10 and 20 °C, showed a reduction of sensitivity but no changes in R_max_ after 1 hour of hypoxia under CEL and BHTK solutions, suggesting a reduction of sensitivity to an adrenergic-induced vasoconstrictor response. Regarding STH-1 solution, the sensitivity reduced after 20 °C but not after exposure to 10 °C and R_max_ increased after exposure to 10 °C when compared to control conditions (Krebs at 37 °C). However, comparing concentration-response curves to phenylephrine, after exposure to STH-1 solution at 10 °C and 20 °C, an increase in sensitivity and R_max_ was observed, suggesting an increased reactivity to adrenergic vasoconstrictors.

Endothelial damage (Figure 8) reinforced such findings. CEL and BHTK solutions increased R_max_ and sensitivity after endothelial removal at both temperatures but these changes were enhanced after exposure to 10 °C. However, after exposure to STH-1 solution, sensitivity and R_max_ were enhanced after exposure to 20 °C. Regarding coronary arteries, only a few reports presented effects of preservation solutions. Perrault et al.^[10]^ investigated the effects of preservation solution on coronary relaxation, showing that only CEL better preserved endothelial dependent relaxation. Yang and He^[11]^ reviewed the effects on coronary arteries by preservation solutions, suggesting that they might have detrimental effects on the endothelium, mainly related to solutions containing high potassium concentrations. Then, if the rings exposed to 10 °C have a damaged endothelium when temperature increases, the reactivity to vasoconstrictors will increase dramatically, which might impair blood flow and ventricular ejection.

Our findings suggest that CEL and BHTK solutions reduced vascular reactivity to phenylephrine in the aortic rings after exposure to 20 °C and 10 °C with intact endothelium. When the endothelium was removed, an increase in R_max_ and sensitivity occurred after exposure to 10 °C, suggesting that, at this low temperature, a dysfunctional endothelium will provide conditions for an increased vasoconstriction. Regarding the STH-1 solution, an increased reactivity to phenylephrine was observed, mainly after exposure to 10 °C. After endothelium removal, an enhanced reactivity was also observed after exposure to 20 °C. Once again, a dysfunctional endothelium will provide conditions for an increased vasoconstriction to occur.

We should emphasize that solutions that reduce vascular tone may have a better profile for myocardial perfusion and, consequently, cardiac recuperation. In addition, the reduction of conductance arteries tone reduces the afterload during ventricular ejection. In this context, this study showed that CEL and BHTK solutions reduced vascular tone, pointing towards an increase of blood flow and ventricular ejection, increasing cardioprotection.

### Limitations of the Study

In this study, the endothelial factors involved in the changes described here were not investigated. This evaluation is under way. However, due to the original results presented here and the enormous amount of protocols that are necessary to study the role of several endothelial factors, this work was presented. The fact that patients undergoing surgical procedures usually have atherosclerosis and hypertension, conditions that might damage the endothelium, this will enhance the vascular smooth muscle contractility and, consequently, are enough to justify the presentation of this study.

## Conclusion

In summary, our results show that hypoxia for 1 hour did not damage the endothelial function with the solutions used here but preservation under different temperatures might produce harmful consequences. However, CEL and BHTK solutions reduced vascular reactivity to phenylephrine at 37, 20 and 10 °C. Moreover, this decreased vascular reactivity to phenylephrine correlated with the lower potassium concentration, which is lower in these solutions. It is important to emphasize that, although CEL and BHTK solutions were the ones that reduced the vascular tone in aortic rings, only CEL solution showed a better myocardial protection response in isolated rat hearts submitted to cold static ischemia^[12]^. In fact, solutions that reduce the vascular response to vasoconstrictors, a necessary condition to increase blood flow and reduce afterload, seem to have a better profile for better myocardial perfusion and protection and, consequently, for cardiac recovery. We must emphasize that cardiac surgeries are usually performed in patients with atherosclerotic disease or other conditions accompanied by endothelial dysfunction. In such conditions, the selection of myocardial protection procedures submitted to cold static ischemia needs special attention regarding adequate composition and temperature of the solution.

**Table:** 

Authors' roles & responsibilities	
PRB	Substantial contributions to the conception or design of the work; or the acquisition, analysis, or interpretation of data for the work; drafting the work or revising it critically for important intellectual content; agreement to be accountable for all aspects of the work in ensuring that questions related to the accuracy or integrity of any part of the work are appropriately investigated and resolved; finla approval of the version to be published
DVV	Substantial contributions to the conception or design of the work; or the acquisition, analysis, or interpretation of data for the work; drafting the work or revising it critically for important intellectual content; agreement to be accountable for all aspects of the work in ensuring that questions related to the accuracy or integrity of any part of the work are appropriately investigated and resolved; final approval of the version to be published
MRS	Substantial contributions to the conception or design of the work; or the acquisition, analysis, or interpretation of data for the work; drafting the work or revising it critically for important intellectual content; agreement to be accountable for all aspects of the work in ensuring that questions related to the accuracy or integrity of any part of the work are appropriately investigated and resolved; final approval of the version to be published
MLL	Substantial contributions to the conception or design of the work; or the acquisition, analysis, or interpretation of data for the work; drafting the work or revising it critically for important intellectual content; agreement to be accountable for all aspects of the work in ensuring that questions related to the accuracy or integrity of any part of the work are appropriately investigated and resolved; final approval of the version to be published
